# Identification of Specific Nuclear Genetic Loci and Genes That Interact With the Mitochondrial Genome and Contribute to Fecundity in *Caenorhabditis elegans*

**DOI:** 10.3389/fgene.2019.00028

**Published:** 2019-02-04

**Authors:** Zuobin Zhu, Xiaoxiao Han, Yuechen Wang, Wei Liu, Yue Lu, Chang Xu, Xitao Wang, Lin Hao, Yuanjian Song, Shi Huang, Joshua D. Rizak, Ying Li, Conghui Han

**Affiliations:** ^1^Department of Genetics, Research Facility Center for Morphology, Xuzhou Medical University, Xuzhou, China; ^2^Center of Reproductive Medicine, Shanghai First Maternity and Infant Hospital, Tongji University School of Medicine, Shanghai, China; ^3^Medical Technology College, Xuzhou Medical University, Xuzhou, China; ^4^Department of Clinical Medicine, Xuzhou Medical University, Xuzhou, China; ^5^Department of Urology, Xuzhou Central Hospital, Xuzhou, China; ^6^School of Life Sciences, Xiangya Medical School, Central South University, Changsha, China; ^7^R.A.S. Innovation, Regina, SK, Canada

**Keywords:** *C. elegans*, fecundity, nuclear, mitochondria, epistasis, genetics, genes

## Abstract

Previous studies have found that fecundity is a multigenic trait regulated, in part, by mitochondrial-nuclear (mit-n) genetic interactions. However, the identification of specific nuclear genetic loci or genes interacting with the mitochondrial genome and contributing to the quantitative trait fecundity is an unsolved issue. Here, a panel of recombinant inbred advanced intercrossed lines (RIAILs), established from a cross between the N2 and CB4856 strains of *C. elegans*, were used to characterize the underlying genetic basis of mit-n genetic interactions related to fecundity. Sixty-seven single nucleotide polymorphisms (SNPs) were identified by association mapping to be linked with fecundity among 115 SNPs linked to mitotype. This indicated significant epistatic effects between nuclear and mitochondria genetics on fecundity. In addition, two specific nuclear genetic loci interacting with the mitochondrial genome and contributing to fecundity were identified. A significant reduction in fecundity was observed in the RIAILs that carried CB4856 mitochondria and a N2 genotype at locus 1 or a CB4856 genotype at locus 2 relative to the wild-type strains. Then, a hybrid strain (CNC10) was established, which was bred as homoplasmic for the CB4856 mtDNA genome and N2 genotype at locus 1 in the CB4856 nuclear background. The mean fecundity of CNC10 was half the fecundity of the control strain. Several functional characteristics of the mitochondria in CNC10 were also influenced by mit-n interactions. Overall, experimental evidence was presented that specific nuclear genetic loci or genes have interactions with the mitochondrial genome and are associated with fecundity. In total, 18 genes were identified using integrative approaches to have interactions with the mitochondrial genome and to contribute to fecundity.

## Introduction

Fecundity is a complex trait that determines the potential of a species or population to reproduce. The genetic architecture underlying the successful production of offspring is usually related to nuclear genetic variations, such as single nucleotide polymorphisms (SNPs) (Gentiluomo et al., 2017; Mishima et al., 2018). However, there is an accumulation of evidence that suggests a link between mitochondrial genetic variants or mitochondrial dysfunction and fecundity. Normally, mitochondria occupy up to 20% of a eukaryotic cell volume and function to provide the primary energy source for eukaryotic cells (Brand, 2014). Mitochondria are also essential organelles for growth and development. For example, daughter cells of stem-like cells have been reported to inherit younger mitochondria and to exhibit higher stem-like activity, suggesting that mitochondria play a central role in cell proliferation (Katajisto et al., 2015). Mitochondria dysfunction, on the other hand, is known to lead to low sperm quality (Amaral et al., 2013), egg maturation or fertilization abnormalities, embryo loss (Seli, 2016; Tsai and St, 2018) and premature ovarian failure (Pagnamenta et al., 2006). This indicates that multiple elements of mitochondria function/dysfunction influence fecundity.

There are several lines of evidence for mitochondrial-nuclear (mit-n) genetic coevolution (Osada and Akashi, 2012; Levin et al., 2014; Sunnucks et al., 2017). While there are over 1,000 proteins involved in the function of mitochondria, only 13 of these proteins are encoded in mitochondrial DNA (Vogtle et al., 2017). The other proteins are encoded in the nuclear genome and are imported into the mitochondria, highlighting the interdependence of mitochondrial and nuclear genetics. In fact, many mit-n interactions have been elucidated with respect to biological fitness for many species (Meiklejohn et al., 2013; Zhu et al., 2014, 2015b).

However, it has not been clearly described how specific nuclear loci interact with mitochondrial genetics (mit-n genetic interactions) and result in lower fitness in some organisms and contribute to the quantitative trait of fecundity. Genetic incompatibility (Dobzhansky-Muller incompatibility) is a model of reproductive isolation that suggests hybrid in-viability or sterility is caused by incompatible interactions between genetic loci that diverged in two distinct species (Orr and Turelli, 2001). Generally, these reproduction isolation models caused by deleterious interactions of alleles are advantageous or neutral in their own genetic backgrounds (Orr and Presgraves, 2000; Seidel et al., 2008). However, these models have other important implications when fertility falls in a population. In the past two decades, scientists have discovered several genetic loci causing hybrid sterility or in-viability (Lee et al., 2008; Chen et al., 2017; Koide et al., 2018), but the major effects of these genes do not completely explain fecundity. Moreover, these studies did not identify the effects of mit-n genetic interactions on the quantitative characteristics of fecundity. As such, identifying more incompatible loci between nuclear and mitochondrial genomes is vital for understanding the complex quantitative trait of fecundity.

*Caenorhabditis elegans* has been widely used as a model for fecundity research (Cutter, 2004; McJunkin and Ambros, 2014; Bundus et al., 2018; Pradhan et al., 2018). A comparison of two nematode strains [N2 wildtype isolates (Bristol strain) and CB4856 wildtype isolates (Hawaii strain, CB4856)] identified a p.A12S amino acid substitution in the COX1 catalytic subunit of mitochondrial respiratory complex IV (Dingley et al., 2014). The Hawaii (CB4856) wild-type isolate had significant lower brood sizes than the N2 wild-type isolates (Andersen et al., 2014, 2015). Our previous work suggested that the brood size of *C. elegans* was explained only in part by nuclear genes (Yuan et al., 2014). In this study, the role of mitochondria in fecundity was investigated to identify specific nuclear genetic loci and genes that interact with the mitochondrial genome and contribute to fecundity in *C. elegans*. To accomplish this, recombinant inbred advanced intercross lines (RIAILs) derived from the two wild-type strains of *C. elegans* (N2 and CB4856) (Rockman and Kruglyak, 2009; Andersen et al., 2015) and hybrid strains with CB4856 mitochondria and specific nuclear genetic loci were used to further elucidate mit-n genetic interactions with respect to fecundity because these two wild-type strains have abundant genetic variations in both their mitochondrial (Dingley et al., 2014) and nuclear genomes (Solorzano et al., 2011). In constructing the RIAILs, F1 to F10 progenies were randomly single-pair mated to maximize random recombination and to form genetic diversity in the offspring population. The offspring were then inbred up to 20 generations to generate the final panel of strains, resulting in plenty of recombination between the mitotype and nuclear genomes of these two strains.

Then, a new integrative approach that includes gene association mapping and a double correlation method (Zhu et al., 2015a) was applied to analyze the genomes of the RIAILs because complex traits, like fecundity, are known to be polygenic and display quantitative genetic variations (Bloom et al., 2013). Current methods to analyze genomes are usually focused on identifying individual loci with large effects on traits. These approaches include linkage analysis or quantitative trait loci (QTL) mapping and genome-wide association studies (GWAS) (Manolio et al., 2009; Eichler et al., 2010) and have been implored successfully in recent years. For example, epistatic interactions among alleles have been found to shape genomic diversity (Rockman et al., 2010; Thompson et al., 2015) and to account for missing heritability (Edwards et al., 2014; Woo et al., 2017). However, extremely large sample sizes are necessary, in most cases, for GWAS to detect weak loci effects (Ripke et al., 2013). These challenges require a new approach to accurately evaluate mit-n genetic interactions and to elucidate candidate genes important to fecundity.

To address this, a set of 1,454 SNP markers spanning 98.6% of the chromosomes previously genotyped in the RIAILs (Rockman and Kruglyak, 2009) and two QTLs on chromosomes IV and X previously found in the RIAILs to be linked with fecundity in *C. elegans* (Andersen et al., 2014, 2015) were used as templates to focus the search for candidate genes associated to fecundity. Then, biochemical experiments focused on mitochondrial function were performed to provide evidence that loci linked with fecundity have interactions with mitochondrial function in addition to mit-n genetic interactions.

## Materials and Methods

### Strains and Media

Eighty-three N2-CB4856 RIAILs were kindly gifted by L. Kruglyak from the Department of Biological Chemistry, and Howard Hughes Medical Institute, University of California. *C. elegans* were cultivated at 20°C on nematode growth medium (NGM) and seeded with *E. coli* OP50.

### Generation of Hybrid Worm Strains

#### (A) Generation of Homoplasmic Mitochondrial Genome Hybrids

The CN30 strain was obtained by crossing three N2 males with a CB4856 hermaphrodite and then repeatedly crossing the offspring with N2 males up to 20 generations. The NC30 strain was obtained by crossing three CB4856 males with a N2 hermaphrodite and then repeatedly crossing the offspring with N2 males up to 20 generations.

#### (B) Generation of Homoplasmic Mitochondrial and Altered Nuclear Genome Hybrids

The CNC10 strain was generated by crossing QX117 males (gifted by L. Kruglyak) with a CB4856 hermaphrodite and then repeatedly crossing the offspring with CB4856 males. The offspring with a N2 genotype at locus 1 were selected and then mated with a CB4856 hermaphrodite. The above steps were repeated for at least 10 generations. The strain that carried the CB4856 genotype at locus 1 was addressed as CCC10. Note: The QX117 strain contained the N2 genotype at locus 1 and CB4856 genotype at locus 2. There were several breakpoints on both sides (within range of 200 kb) of locus 1.

Every strain used in this work were inbred at least 10 generations and sequence validated by PCR to ensure they had the corresponding nuclear and mitochondria background.

### HAC Calculation

The CB4856 allele content (CAC) of each strain was calculated as the number of CB4856 alleles carried by the RIAIL divided by the number of SNPs examined. The 1,454 SNPs genotype data for the RIAILs were downloaded from a previously published dataset (Rockman and Kruglyak, 2009).

### Association Mapping

The PLINK software package (1.90) with the quantitative trait association option was used to search for SNPs linked with fecundity and mitochondria. The quantitative trait association approach uses a permutation procedure to control for the non-independence of individuals. The analysis of phenotype–genotype association was a standard regression of phenotype on genotype that ignores family structure (Purcell et al., 2007). *Q*-value estimation for false discovery rate (FDR) controls was analyzed using the R package ‘*q*-value’ (Storey and Tibshirani, 2003).

### Lifespan Brood Size Measurement

All strains were synchronized by transferring 10–20 adult worms to a fresh dish and allowing them to lay eggs for 3 h. Afterward, the worms were picked out. Twenty L4 stage worms were placed into 20 dishes, respectively. Then they were allowed to lay eggs each day in a new dish until no more eggs were laid. The eggs in each dish were allowed to develop for 2 days before being counted.

### RNAi Assays

RNAi was performed essentially as described (Zhu et al., 2015a). L1 worms placed on the NGM plates seeded with the HT115(DE3) bacteria were transformed with the L4440 vector containing a fragment corresponding to the target gene. The primers of RNAi fragments were listed in Supplementary Table S4. For negative controls, the L1 worms were fed with the HT115 bacteria carrying the L4440 vector with no gene fragment. For positive controls, the L1 worms were fed with the HT115 bacteria carrying the L4440 vector with cyc-1 fragment.

### Hatching Rate Measurement

Twenty L4 stage worms were placed into 20 dishes, respectively. Then they were allowed to lay eggs for 8 h in a new dish until no more eggs were laid. The eggs in each dish were counted and the larvae were counted 2 days after hatching.

### Determination of the Origin of Mitochondria

Mitochondria were isolated as previously described (Jonassen et al., 2002). Two SNPs in the mtDNA between the N2 and CB4856 strain were determined by PCR (Zhu et al., 2015b). SNP1 is located at site 2038 in the N2 mitochondrial genome (LK928807) (AGAATGATTTACGTTACCA/TTATTTTTTTGATTTT, A = N2, T = CB4856) and SNP2 is located at site 3444 in the mitochondrial genome (LK928807) (ATTTCTTTATTTACC/GTTGTT TTTAACATTAT, C = N2, G = CB4856). The nuclear DNA was extracted from worms and amplified with SNP1 and SNP2 primers (SNP1, F, ATAACACCCTTAAATTCCTC, R, CTAAC TCCCTTTCACCTTC; SNP2, F, CAACTAACGAGTTCATAAAGCAA, R, GACCTCCTCTA CAAAGAAGAAATAA). The origin of mitochondria in the 83 strains used in this study was determined by PCR.

### Measurement of Relative mtDNA Content

Quantitative real-time PCR was used to determine the relative copy number of mtDNA to nuclear DNA (Artal-Sanz and Tavernarakis, 2009; Zhu et al., 2015b). The relative levels of mtDNA were determined in synchronous populations of L4 stage larvae. Primers specific to mtDNA (F, TGGAACTCTGGAGTCACACC and R, CATCCTCCTTCATTGAACGG) were used. The results were normalized to nuclear DNA (F, TGGAACTCTGGAGTCACACC, R, CATCCTCCTTCATTGAACGG). Quantitative real-time PCR was performed using the SYBR Green Supermix (Bio-Rad: cat #170-8882AP). Every experiment was repeated three times.

### Measurement of Adenosine Tri-Phosphate (ATP) Levels

ATP concentration in the L4 stage worms was measured by an ATP bioluminescent assay kit. The number of worms was counted (about 300 adults). The worms were then washed with M9 buffer containing 0.1% Tween-20 three times to remove the *E. coli* OP50. All, but 500 μL, of M9 buffer was removed from the worm pellet. Then the worms were lysed as previously described (Tanji et al., 2013). Finally, ATP was measured using a luciferase based assay, as per the manufacturer’s described protocol (Sigma, product number FL-AA). The ATP concentration was normalized to the number of worms. The stage of worms used and the time of the lysis conditions for worms are important to maintain the stability of the experimental data. All the worm strains used in this study were tested synchronously and each experiment was repeated three times. The final ATP concentration reported was the mean of the normalized ATP concentration per worm.

### Measurement of Reactive Oxygen Species (ROS) Levels

Dichlorofluorescin diacetate (DCFDA) was used to determine the level of intracellular ROS in *C. elegans*. L4 stage worms were washed three times with M9 buffer and then stained with DCFDA solution at a final concentration of 50 μM DCFDA for 30 min at 20°C. Twenty randomly selected worms were transferred onto microscope slides coated with 3% agarose and anesthetized with 2% sodium azide. Worms were photographed immediately using a fluorescence microscope (100× magnifications and 200 s exposure time). The area of the images was calculated by the software Image-Pro Plus.

### Statistical Methods

Spearman and Pearson analyses were performed using GraphPad Prism 5. Normalized gene expression data for the RIAILs were obtained from published datasets (Rockman and Kruglyak, 2009). The correlation between gene expression and fecundity or mitotype was analyzed using the Significance Analysis of Microarrays (SAM) software, which utilizes a modified *t*-test statistic with 1,000 sample-label permutations to evaluate statistical significance (Tusher et al., 2001). Estimation for FDR control was done using the R package ‘*q*-value’ function (Storey and Tibshirani, 2003). DAVID bioinformatics resources consist of an integrated biological knowledgebase and analytic tools aiming at systematically extracting biological meaning from large gene/protein lists (Huang et al., 2009). The functional classification tool of DAVID generated a gene-to-gene similarity matrix based on shared functional annotation using over 75,000 terms from 14 functional annotation sources. The novel clustering algorithms classified highly related genes into functionally related groups. Then, the DAVID tool predicted related functional pathways of the genes that matched the Kyoto Encyclopedia of Genes and Genomes (KEGG).

## Results

### Multiple Loci Affect Fecundity of *C. elegans*

**FIGURE 1:**
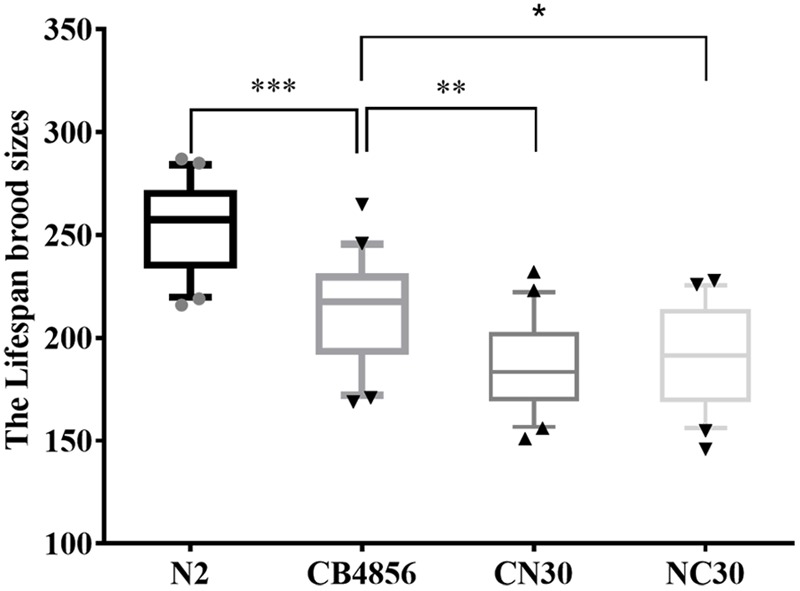
Lifetime fecundity of wild type and hybrid *C. elegans*. The number of offspring of *C. elegans* was counted for more than 8 days until there were no more eggs laid. Data shown are the median (10–90% percentile). ^∗^*P* < 0.05, ^∗∗^*P* < 0.01, ^∗∗∗^*P* < 0.001, Student’s *t*-test.

A significantly lower fecundity of CB4856 worms has been observed relative to N2 worms (Andersen et al., 2014, 2015). Here, two ‘mito-nuclear’ strains of *C. elegans* were generated by placing mtDNA from strains of CB4856 onto controlled nuclear backgrounds of N2 (CN30) and by placing mtDNA from strains of N2 onto controlled nuclear backgrounds of CB4856 (NC30) to examine whether a reduction in fecundity was caused by variations in mitochondrial genetics. Comparison of the two strains with the same nuclear background (N2 and CN30) found the fecundity of CN30 to be significantly lower than the wild-type N2 strain (Figure 1). This suggested that the reduction in fecundity may be caused by the variation in mitochondria of CB4856. Comparison of the two strains with the same mitochondria background (N2 and NC30) found the fecundity of NC30 was significantly lower than the wild-type N2 strain. This suggested that the reduction in fecundity may be caused by the variation in the nuclear genetic components of CB4856. In summary, both hybrid strains (CN30 and NC30) had significantly lower fecundity than the wildtype strains. These results indicated that the reduction in fecundity was mainly caused by the incompatibility of mitochondrial and nuclear SNPs in the nematodes.

**FIGURE 2:**
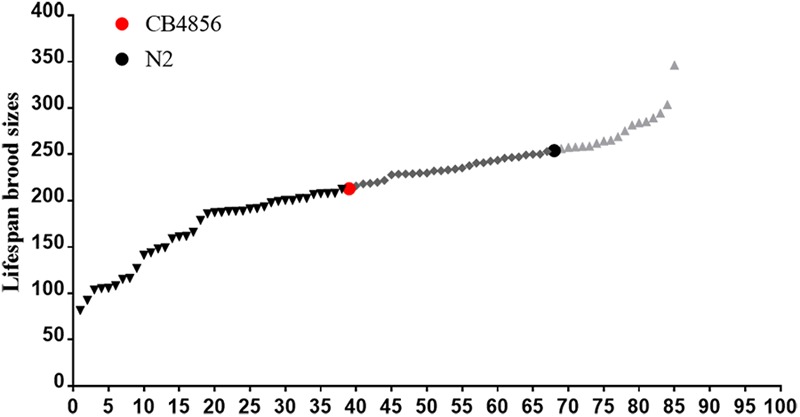
Fecundity of 83 N2-CB4856 recombinant inbred advanced intercross lines (RIAILs). The brood sizes for each of the 83 RIAILs (*x*-axis) provided by Dr. Kruglyak.

To determine the difference between the genetic architecture related to fecundity of the N2 and CB4856 strains, brood sizes of 83 N2-CB4856 recombinant inbred advanced intercross lines were quantified (Figure 2). Thirty-eight of the RIAILs had lower brood sizes comparable to CB4856, 17 had higher brood sizes comparable to N2, and 28 had intermediate brood sizes (Figure 2). In 192 RIAILs strains (with determined nuclear and mitochondrial genotypes) published previously (Andersen et al., 2015), 61 of the RIAILs had lower brood sizes comparable to the CB4856 strain, 46 had higher brood sizes comparable to N2, and 85 had intermediate brood sizes (Supplementary Table S1). The excess of lower or higher fecundity than the wild-type strains suggested that there must be some genetic incompatibility in the both RIAILs. This finding was consistent with the above results (Figure 1).

### The Relationship Between Mitochondria and Fecundity in *C. elegans*

**FIGURE 3:**
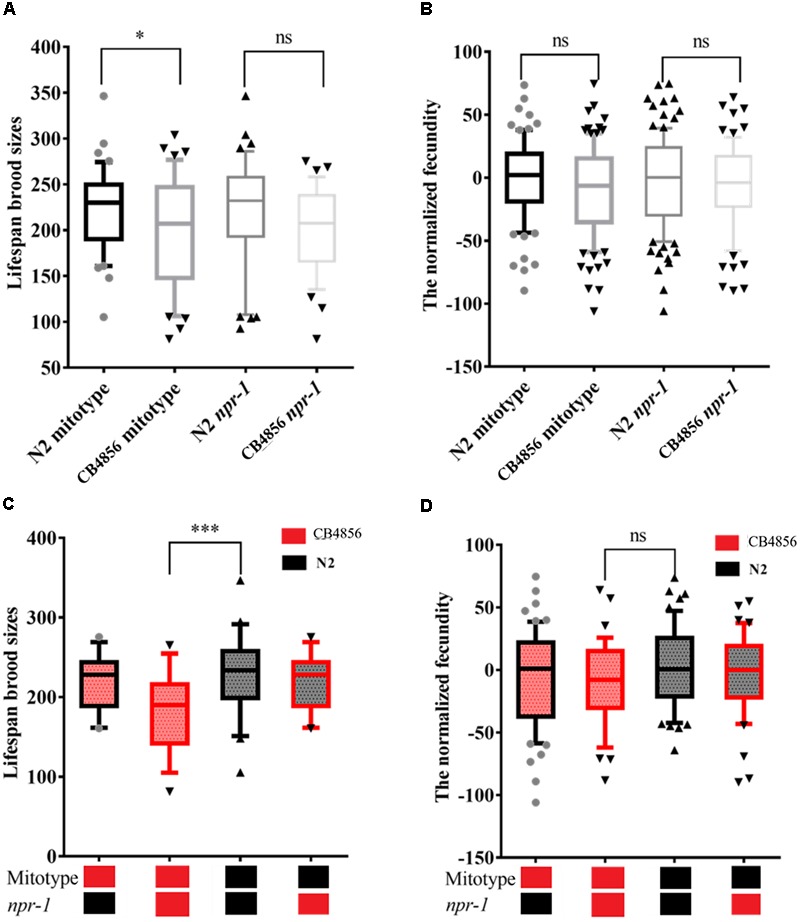
The effect of N2 and CB4856 alleles of the *npr-1* gene and mitochondrial type on fecundity. Box plots show a summary of phenotype data of nematode RIAILs that carried different mitochondria or genotypes of *npr-1* (Bristol in black (N2) and Hawaii in red (CB4856)). The effect of N2 and CB4856 alleles of the *npr-1* gene and mitochondrial type on fecundity in the 83 RIAILs **(A)** and the 192 RIAILs published by Andersen et al. **(B)**. The interaction effect between the *npr-1* gene and mitochondrial type on fecundity in the 83 RIAILs **(C)** and the 192 RIAILs published by Andersen et al. **(D)**. Data shown are the median (10% - 90% percentile). ^∗^*P* < 0.05, ^∗∗∗^*P* < 0.001, Student’s t-test.

The mitochondria type of the 83 strains was then determined by PCR. This PCR analysis obtained the same results as a previous report (Doroszuk et al., 2009). The RIAILs that carried the CB4856 mitochondria were found to have a lower brood size than those that carried the N2 mitochondria (Figure 3A). Although replicate analysis of the published datasets (Andersen et al., 2015) did not strongly confirm this result (*P* > 0.05), these findings showed similar trends in fecundity (Figure 3B).

### The Relationship Between the *npr-1* Polymorphism and Fecundity in *C. elegans*

*npr-1* is known to have large physiological and behavioral effects (Reddy et al., 2009; Bendesky et al., 2011; Andersen et al., 2014). Here, the effects of *npr-1* on fecundity were examined (Figure 3A,B). SNPs co-segregating with *npr-1*, defined as SNPs closest to *npr-1*, were not found linked with fecundity in the two independent datasets (A) the fecundity data of the 83 strains and B) the 192 strains published by Andersen et al. (2015). The fecundity of RIAILs that carried CB4856 mitochondria and CB4856 *npr-1* was significantly lower than those that carried N2 mitochondria and N2 *npr-1* (Figure 3C). This result was confirmed by replicate analysis using previously published datasets (Andersen et al., 2015) and was found to be less dramatic than the previous dataset (Figure 3D, *P* < 0.05, Student’s *t*-test, one tailed). Although variation in mitochondria or *npr-1* is known to cause phenotypic differences in a multiple of other traits, these variations are not solely responsible for the large phenotypic differences in fecundity.

### SNPs Linked With Fecundity

Single nucleotide polymorphisms that may be linked with fecundity were searched for using the quantitative trait association option of the PLINK software to estimate whether other nuclear loci have a potential role in fecundity. In the 83 strains, 269 SNPs (among the set of 1,454 SNPs examined) were found to be linked with fecundity by a logistic regression test of the 83 RIAILs. Among these SNPs, 7 were located on chromosome II, 15 were located on chromosome III, 169 were located on chromosome IV, 77 were located on chromosome V (*P* < 0.05, FDR < 10%, Supplementary Table S2). Replicate analysis of the previously published datasets including the 192 strains (Andersen et al., 2015) found 241 SNPs to be linked with fecundity. Among these SNPs, 3 were located on chromosome II, 33 were located on chromosome III, 124 were located on chromosome IV, and 82 were located on chromosome V (FDR < 10%, Supplementary Table S2). Overall, 115 SNPs were linked with fecundity in the 83 strains and the 192 strains: 2 were located on chromosome III, 113 were located on chromosome IV (Supplementary Table S2). These results suggested a causal link between these regions on the chromosomes and the fecundity trait. Furthermore, all CB4856 genotypes of the 113 SNPs found on chromosome IV showed negative correlation with fecundity (Supplementary Table S2). Since most CB4856 types of SNPs were associated with reduced fecundity, the strains that carried higher CB4856 allele content (CAC) was expected to have lower fecundity.

The 1,454 SNPs were used to calculate the CB4856 allele content (CAC) of each RIAIL. The HAC was defined as the number of CB4856 alleles divided by the number of SNPs examined. A large variation in HAC was found (∼0.09 to ∼0.8) among the RIAILs (Supplementary Table S1). There was no relationship between HAC and fecundity (Supplementary Figures S1A,B) and there was no difference in HAC between the CB4856 mitotype strains and N2 mitotype strains (Supplementary Figures S1C,D). These results indicated that the difference in fecundity between the CB4856 mitotype strains and N2 mitotype strains was not simply influenced by the number of CB4856 alleles.

### The Effect of Epistasis Between Nuclear SNPs and Mitotypes on Fecundity

Single nucleotide polymorphisms linked with mitotype were analyzed using the case/control association test of the PLINK software (Purcell et al., 2007). The analysis showed that a total of 558 SNPs representing 38.4% (558/1454) of the genome were linked with mitotype and were located on the 6 chromosomes in *C. elegans* (Supplementary Table S2). Further analysis showed that there were 67 SNPs linked with mitotype among the 115 SNPs linked with fecundity (65/115 vs. 558/1454, *P* < 0.01, Fisher Exact Test, two tailed) (Supplementary Table S2). These results indicated that there were significant epistatic effects between nuclear and mitochondria SNPs on the fecundity trait.

**FIGURE 4:**
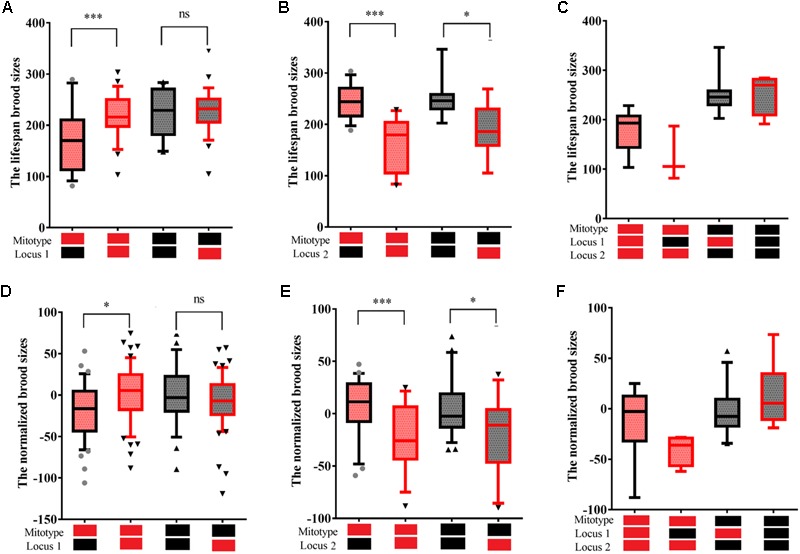
Fecundity of RIAILs *C. elegans*. Box plots show summary phenotype data of the RIAILs that carried different mitochondria or genotypes at one of two loci on the chromosomes [Bristol in black (N2) and Hawaii in red (CB4856)]. Lifespan brood sizes were counted by standard laboratory agar plate assays **(A–C)**. Normalized brood sizes of the RIAILs *C. elegans* were counted by high-throughput assays of nematodes grown in 96-well microtiter plates **(D–F)** and from data downloaded from previously published dataset (Andersen et al., 2015). Data shown are the median (10–90% percentile). ^∗^*P* < 0.05, ^∗∗∗^*P* < 0.001, Student’s *t*-test.

To prevent interference of co-segregating SNPs, PLINK was used with a pairwise LD calculated for SNPs located within 200 kb. Twelve haplotype blocks were found to be linked with fecundity (Supplementary Table S3). Among the 12 haplotype blocks, two blocks located on a 110 kb extent of chromosome III were found with high LD pairwise *R*^2^ (>0.7) and to have similar phenotypic effects on fecundity. These two blocks have been referred to as locus 1 in subsequent mentioning. Locus 1 was found to have a significant incompatibility with mitotype. Ten haplotype blocks were also found on an extent of 4 Mb of chromosome IV and to have similar phenotypic effects of fecundity. This 10 block region is subsequently referred to as locus 2. To reduce interactions among the 10 blocks, the RIAILs that carried the same genotype within the 10 blocks were further analyzed. The N2 genotype of locus 1 was found to significantly reduce fecundity for RIAILs (83 strains) with a CB4856 mitochondrial background but not a N2 mitochondrial background (Figure 4A). The CB4856 genotype of locus 2 was found to significantly reduce fecundity for RIAILs with a CB4856 mitochondrial background. However, this trend was decreased in RIAILs with a N2 mitochondria background (Figure 4B). It was further found that strains that carried the CB4856 mitotype combined with a N2 genotype at locus 1 and a CB4856 genotype at locus 2 had the lowest level of fecundity (Figure 4C). These results were confirmed by using the data set of previously published RIAILs (Andersen et al., 2015) (Figure 4D–F). This suggested that the epistatic interactions between mitochondria and specific nuclear loci were one of the main reasons for the reduction in fecundity.

**FIGURE 5:**
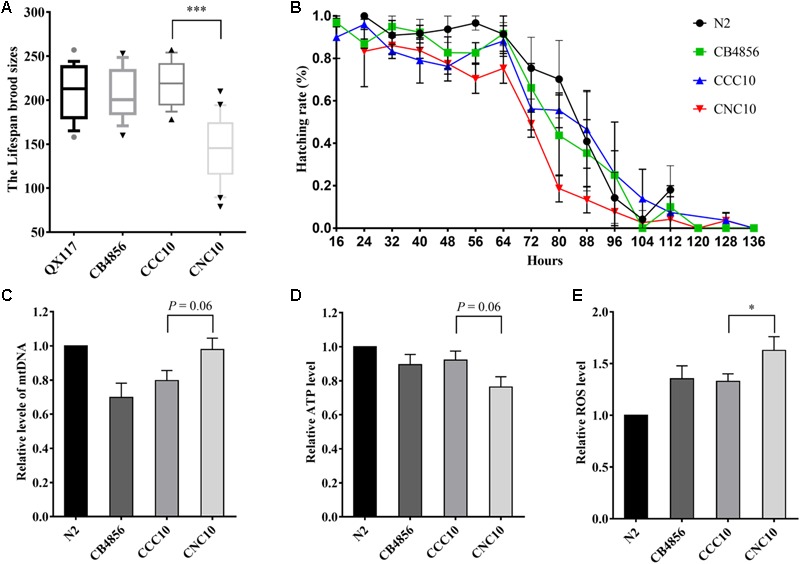
Wild-type and hybrid strain variations in fecundity, hatching rate and functional characteristics of mitochondria. The CNC10 hybrid strain was bred as homoplasmic for the CB4856 mtDNA genome and for N2 genotype at locus 1 in the CB4856 nuclear background. The CCC10 strain was bred as homoplasmic for the CB4856 mtDNA genome and for CB4856 genotype at locus 1 in the CB4856 nuclear background. **(A)** Variation in fecundity. **(B)** Variation in hatching rate. **(C)** The variation in relative level of mtDNA. **(D)** The variation in relative ATP levels. **(E)** The variation in relative ROS levels. Data shown are the median (10–90% percentile) in **(A)** and means ± SEM in **(C–E)**. ^∗^*P* < 0.05, ^∗∗∗^*P* < 0.001, Student’s *t*-test.

Next, a strain, denoted CNC10, that carried CB4856 mitochondria and the whole genome region of the CB4856 genotype except for locus 1, which was of the N2 genotype, and a strain, denoted CCC10, that carried CB4856 mitochondria and the nuclear genome of the CB4856 genotype as a control were generated (see “Materials and Methods” section) to further demonstrate that the incompatibility between a CB4856 mitochondria and the N2 genotype at locus 1 that reduced fecundity was not simply influenced by other genetic factors. The CNC10 strain, compared to CCC10, displayed a significant reduction in fecundity and hatching rate (Figure 5A,B) that was consistent with the above results from the RIAILs (Figure 4C,F).

To study the molecular mechanisms related to the relationship between mit-n incompatibility and fecundity, relative mtDNA genome content of the two wildtype isolates and the two new strains were assessed by quantitative polymerase chain reaction (qPCR) analysis. Interestingly, the CB4856 and CCC10 strains displayed a 20–30% decrease in mtDNA content in comparison to the N2 strain. The CNC10 strain, on the other hand, had a 20% increase in mtDNA content relative to CCC10 (Figure 5C). The ATP content and ROS levels of these strains were further evaluated to assess mitochondrial function/dysfunction. It was found that the CNC10 strain had lower ATP content and higher ROS level than the CCC10 strain (Figure 5D,E).

### Identification of Novel Genes Related to Fecundity

Previously published datasets of normalized gene expressions (Rockman and Kruglyak, 2009) were used to identify novels genes related to fecundity. 2,037 genes were found to be linked with fecundity (*P* < 0.05, FDR < 5%, Supplementary Table S4) in the 83 RIAILs, 6,280 genes linked with fecundity (*P* < 0.05, FDR < 5%, Supplementary Table S4) were found in the previously described RIAILs (Andersen et al., 2015), and 1,581 genes linked to fecundity were found in both the two datasets among 15,888 genes examined (*P* < 0.05, FDR < 10%). The most significantly linked biological pathways for the 1,581 genes found in the Kyoto Encyclopedia of Genes and Genomes (KEGG) were related to glycerolipid metabolism and RNA transport (Supplementary Table S5).

These results suggest that the genetic loci and mitotype affect a complex trait and that a gene linked with the genetic loci, mitotype and fecundity should have a higher probability to be real target genes than those with only a single correlation. In this analysis, 32 genes that correlated with fecundity and mitotype were identified (Supplementary Table S4). Among the 32 genes, there were 18 genes linked with the genetic loci associated with fecundity (Supplementary Table S5). Annotations of the 18 genes were analyzed against the curated GO database for biological processes (GOTERM_BP_FAT) using DAVID (Huang et al., 2009). This analysis found that there was a large number of genes (39%) involved in embryo development ending in birth or egg hatching (Supplementary Table S6). RNAi experiments were carried out in the N2 strain to further confirm the functions of the four selected genes (three were not annotated in the databases, one have been reported associated with fecundity as positive controls). All the four genes showed a significant decrease in brood sizes (Supplementary Table S4).

## Discussion

For many different organisms, healthy nuclear and mitochondrial genome hybrids have demonstrated that phenotypic differences, which affect biological fitness between species or among distant populations within a species, were due to mit-n interactions and mitochondrial functions (Arnqvist et al., 2010; Du et al., 2017). However, it is less understood which regions of the nuclear genome are incompatible with mitochondrial genetic components. In this study, most of the N2-CB4856 recombinant inbred advanced intercross lines of *C. elegans* were found to have intermediate fecundity, as expected. However, approximately half of the strains had lower fecundity than the CB4856 wild-type strain, which had significantly lower fecundity than the N2 wild-type strain. While this result may be related to a specific loci, such as the p.A12S amino acid difference in the mitochondrial protein COX-1, to natural variations in nuclear DNA or to mit-n mismatches, it remains unknown which regions of the chromosomes have mit-n mismatches and how these factors jointly affect fecundity.

The genetic component of a complex quantitative trait is usually explained as a result of nuclear genome variations, such as a SNPs (Ripke et al., 2013). A good example of this is the laboratory-derived variation in *npr-1*, which has broad pleiotropic effects in *C. elegans* (Reddy et al., 2009; Bendesky et al., 2011; Andersen et al., 2014). However, this polymorphism of *npr-1* was found to have no significant effect on fecundity in this study. Moreover, there was no significant interaction found between *npr-1* genotype and the mitotypes involved in fecundity, which suggested there must be some other loci with a major effect that influences the fecundity trait.

In this study, 115 SNPs linked with fecundity were searched using a genome-wide association mapping technique and found that the CB4856 genotype in nearly all of the SNPs (113/115) had a negative correlation with fecundity. This suggests that strains that carried higher CB4856 alleles would be expected to have lower fecundity if the variations of fecundity were mainly caused by variations in their chromosomes. However, there was no relationship between CB4856 content and fecundity found in this study. The hybrid strain of *C. elegans* with the CB4856 mitochondria in the N2 nuclear background was found to have reduced median fecundity relative to the strain with CB4856 mitochondria and CB4856 nuclear background. These results indicated that the quantitative trait of fecundity in a population was explained, in part, by mit-n compatibility/incompatibility. An earlier study considered that a linkage disequilibrium (LD) analysis should extend only a few kilo bases (KB) from SNPs in the genome (Kruglyak, 1999). However, SNPs closely linked over distances, to the extent of 170 kb, that contribute to gene conversion events or recombination events are clustered and form relatively large separate haplotype blocks (Patil et al., 2001; Gabriel et al., 2002) and that these recombination haplotype blocks have important implications for human disease or complex traits research (Cardon and Abecasis, 2003; Guo et al., 2017). In this study, 12 blocks were found that were linked with fecundity. A genotype of 2 blocks on chromosome III (base-pair position: 2545513–2655404) and a genotype of 10 blocks on chromosome IV (base-pair position: 9192101–13629783) had strong mit-n interactions related to fecundity in the RIAILs with the CB4856 mitotype, but not with the N2 mitotype. This suggested there is another important genetic diversity between N2 and CB4856 nematodes in their mtDNA. The CB4856 mitochondria appear to be less potent or optimal than the N2 mitochondrial type. It has been reported that the variation of p.A12S amino acid in the CB4856 mitochondria increased mitochondrial matrix oxidant burden and sensitivity to oxidative stress (Dingley et al., 2014). Stronger mit-n epistasis of fecundity in the CB4856 mitotype relative to the N2 background indicated that the effect of mit-n epistasis on mitochondrial functions may be more pronounced for less optimal forms of mitochondria, which is *a priori* expected.

To avoid further interferences of genetic factors, a hybrid strain (CNC10) was generated that carried CB4856 mitochondria and the whole nuclear genome region of CB4856 genotype except at locus 1. Several functional characteristics of the mitochondria were influenced in this hybrid strain and the mean fecundity was reduced by half relative to the control strain. The higher mtDNA content, lower ATP content and higher ROS levels in the CNC10 strain suggested that the incompatibility between CB4856 mitochondria and the N2 genotype at locus 1 reduced fecundity in the strain by influencing mitochondrial function. These results were consistent with a functional effect of mit-n epistasis on mitochondria. Moreover, the finding that the number of SNPs involved in the mit-n compatibility was large, rather than small, is also consistent with the high number of nuclear genes directly involved in mitochondria function (as there are more than 1,000 such genes and many more potential genes that regulate them). This suggests that a number of nuclear genes may have been involved in the mit-n interactions evaluated here.

Candidate genes can be screened by several methods, including linkage studies, prior knowledge of the biological pathway, GWAS and comparative genomics strategies (Zhu and Zhao, 2007). However, the false positive rate of identified genes is usually high for most genome wide approaches. Although increasing the association significance threshold can reduce false positive rates, a lot of real genes might also be missed in these approaches. Combining different approaches can increase the power of the approach to identify candidate genes (Kottapalli et al., 2007; Requena et al., 2017). Few of these approaches, however, are meant to identify genes responsible for a quantitative characteristic of a complex trait. Previously, we established a new integrative method for screening decision-making genes or genes for complex traits to effectively screen candidate genes (Zhu et al., 2015a). In this study, 1,581 genes linked with fecundity were found in two independent datasets using this new integrative method. Of these, the genes regulated by mitotype and genotype in the haplotype blocks linked with fecundity needed to be further screened because mit-n interactions are an important genetic factor for fecundity. This screening found 18 genes linked with fecundity, mitotype and genotype in the two independent datasets analyzed in this study. There are good reasons to believe that genes identified by this method have a higher probability to be real target genes related to fecundity. First, *a priori*, a gene linked with fecundity, mitotype and nuclear genotype should also be linked with mit-n interactions contributing to fecundity. Second, candidate genes were also confirmed by another independent dataset (Andersen et al., 2015) and 39% of these genes had been annotated to the function of regulating embryo development or egg hatching. These results suggested that mit-n epistasis might account for the fecundity traits in some of the *C. elegans* hybrid strains.

Such results on mit-n epistasis may be informative to certain evolutionary puzzles on hybrids. For example, inbreeding in humans was common for small tribal societies in ancient times (Prufer et al., 2014), as might be expected. However, ancient DNA data shows that there was hybridization between modern humans and Neanderthals (Prufer et al., 2014; Fu et al., 2015). Some Neanderthal nuclear gene fragments have been detected in modern humans, but no Neanderthal mtDNA has been detected (mtDNAs in GenBank). This suggests that mit-n epistasis might have played an important role in human evolution. Hybrid humans with Neanderthal mtDNA and modern nuclear locus may have poorer fitness relative to those with modern human mtDNA due to mit-n epistasis and may hence have gone extinct. As such, the results here that mit-n interactions contribute to fecundity may help solve the puzzle of why no trace of Neanderthal mtDNA in modern humans has been found despite the presence of Neanderthal nuclear gene fragments. However, it is noteworthy to mention an alternative explanation of why no trace of Neanderthal mtDNA has been found. It is possible that male Neanderthals engaged in sexual reproduction with female *Homo sapiens*, but that male *Homo sapiens* did not engage in sexual reproduction with female Neanderthals, such that no Neanderthal mtDNA was ever passed to future generations of Neanderthal-human hybrids. Nonetheless, refining the mit-n genetic interactions that affect fecundity further may have a role in understanding the Neanderthal puzzle because, in the case male humans did mate with female Neanderthals, some genetic or phenotypical incompatibility may have prevented female Neanderthal gametes from being fertilized by male *Homo sapiens* sperm cells. Further research is needed to elucidate the happenings of the distant past and whether their legacy can be uncovered in mit-n genetic interactions.

In summary, this study identified two loci in the chromosomes of *C. elegans* that interacted with mitochondria and led to quantitative variations in fecundity. This study also identified 19 genes linked with mitotype, genotype and fecundity. These results suggest the two loci and 19 genes identified are of great value to further research into the relationship between mit-n epistasis and fecundity or other complex traits.

## Author Contributions

ZZ: conceptualization, methodology, visualization, writing, – reviewing, and editing, and funding acquisition. XH: methodology, software, validation, and investigation. YW: investigation, and validation. WL, YLu, and CX: investigation. YLu: investigation. CX: investigation. XW: visualization. LH and YS: formal analysis. YS: formal analysis. SH: resources. JR: writing, – reviewing, and editing. YLi: data curation, writing, – reviewing, and editing, project administration. CH: ideas, formulation or evolution of overarching research goals and aims, writing, – reviewing, and editing, and supervision.

## Conflict of Interest Statement

The authors declare that the research was conducted in the absence of any commercial or financial relationships that could be construed as a potential conflict of interest.

## Supplementary Material

FIGURE S1 Correlations between fecundity and HAC of the RIAILs. Correlations between fecundity and HAC in two datasets (**A**, 83 strains; **B**, 192 strains from Andersen et al., 2015). HAC value in the RIAILs with CB4856 mitotype and N2 mitotype (**C**, 83 strains; **D**, 192 strains from Andersen et al., 2015).

TABLE S1 Fecundity data of each RIAIL strain used in this study.

TABLE S2 SNPs linked with fecundity and mitochondria.

TABLE S3 Marker of the blocks which linked with fecundity.

TABLE S4 Genes linked with fecundity, mitochondria and block which linked with fecundity.

TABLE S5 List of related pathways found in KEGG.

TABLE S6 Functional annotation clustering of the genes linked with fecundity.
