# Supplementary figures and images for: Identification of Specific Nuclear Genetic Loci and Genes That Interact With the Mitochondrial Genome and Contribute to Fecundity in Caenorhabditis elegans
Source: Front Genet. 2019 Feb 4;10:28. doi: 10.3389/fgene.2019.00028 (PMC6369210; doi:10.3389/fgene.2019.00028)

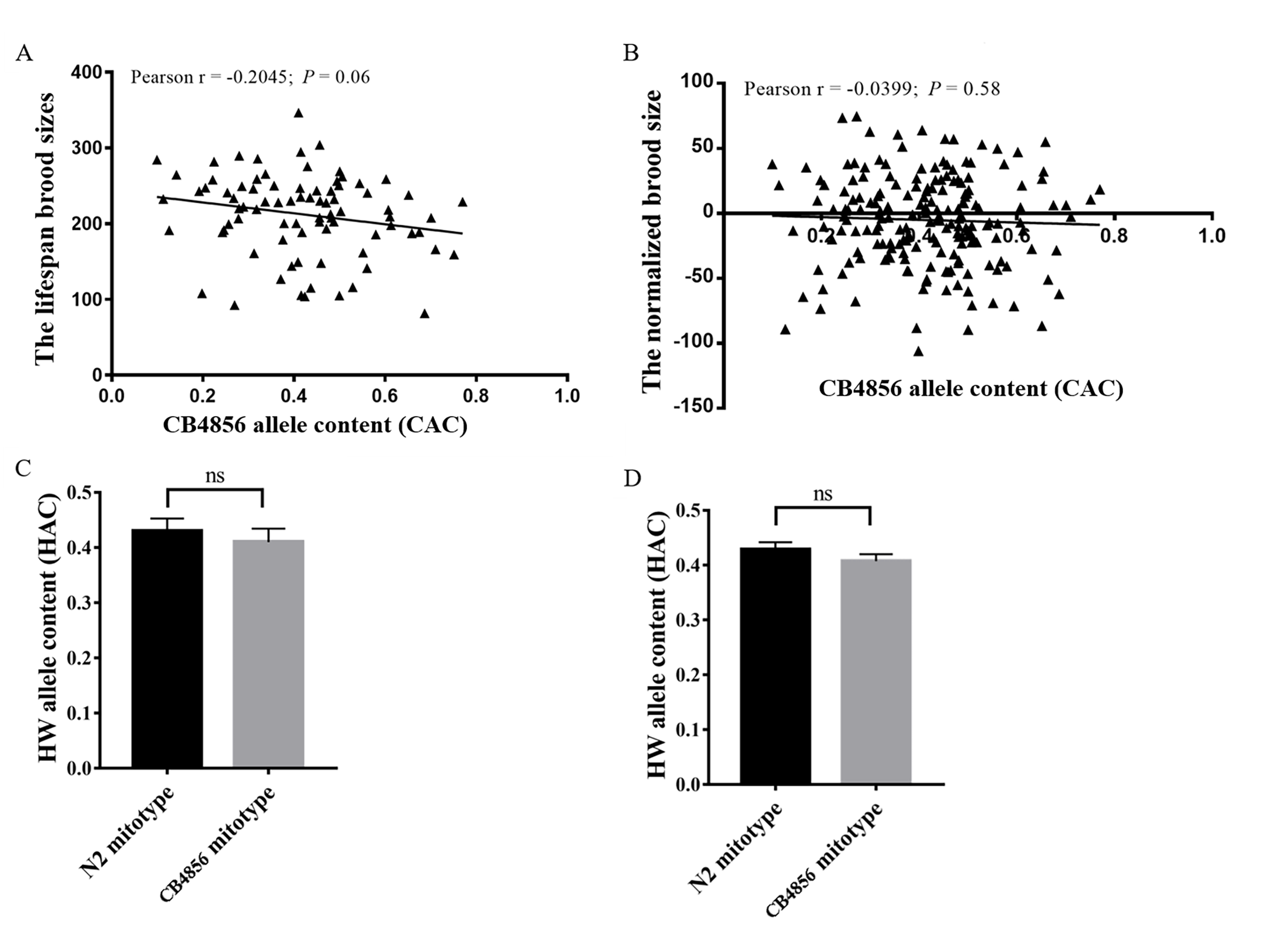

Supplement: FIGURE S1 — Correlations between fecundity and HAC of the RIAILs. Correlations between fecundity and HAC in two datasets (A, 83 strains; B, 192 strains from Andersen et al., 2015). HAC value in the RIAILs with CB4856 mitotype and N2 mitotype (C, 83 strains; D, 192 strains from Andersen et al., 2015). [file Image_1.TIF]
